# A review of neurological side effects of COVID-19 vaccination

**DOI:** 10.1186/s40001-023-00992-0

**Published:** 2023-02-25

**Authors:** Roya Hosseini, Nayere Askari

**Affiliations:** 1 Department of Biology, Faculty of Sciences, Shahid Bahonar University of Kerman, P.O.Box 76135-133, Kerman, Islamic Republic of Iran; 2 Immunoregulation Research Center, Shahed University, Tehran, Islamic Republic of Iran

**Keywords:** SARS Covid-2, COVID-19, Vaccine, Neurological side effects, Thrombosis, Myelitis

## Abstract

Following the COVID-19 virus epidemic, extensive, coordinated international research has led to the rapid development of effective vaccines. Although vaccines are now considered the best way to achieve collective safety and control mortality, due to the critical situation, these vaccines have been issued the emergency use licenses and some of their potential subsequence side effects have been overlooked. At the same time, there are many reports of side effects after getting a COVID-19 vaccine. According to these reports, vaccination can have an adverse event, especially on nervous system. The most important and common complications are cerebrovascular disorders including cerebral venous sinus thrombosis, transient ischemic attack, intracerebral hemorrhage, ischemic stroke, and demyelinating disorders including transverse myelitis, first manifestation of MS, and neuromyelitis optica. These effects are often acute and transient, but they can be severe and even fatal in a few cases. Herein, we have provided a comprehensive review of documents reporting neurological side effects of COVID-19 vaccines in international databases from 2020 to 2022 and discussed neurological disorders possibly caused by vaccination.

## Background

In December 2019, the SARS Covid-2 virus was introduced to the world. A virus that was much more contagious than SARS Covid-1 and spread to different parts of the world in a short time. Following that situation in 2020, the World Health Organization had to declare a global health emergency. This virus is known to cause widespread lung infection and hypoxia [1]. As of November 2022, 630.3 million people have been diagnosed with COVID-19 and 6.58 million deaths worldwide, according to WHO figures [2].

In early 2021, the first vaccines were introduced to stop the pandemic. Also, approximately 68.2% of the world's population has been fully vaccinated against this disease. There are four major strategies for producing COVID-19 vaccines, including nucleic acid-based vaccine (DNA–mRNA), viral vector (replication–non-replication), live inactivated (or attenuated) virus, and protein (spike protein or its subunits). In nucleic acid and adenovirus-based vaccines, fragments of the virus mRNA or genome enter human cells and induce the production of viral proteins [3]. These viral proteins are eventually identified as antigens and stimulate antibody production. In vaccines containing inactive or protein viruses, virus particles and proteins, as antigens, trigger the immune system [4]. As of November 2021, 11 candidate vaccines for COVID-19 have been approved by the World Health Organization for mass vaccination after leaving phase 3 of clinical studies. However, in order to prove the effectiveness of the vaccine in terms of safety and side effects, the implementation of phase 4 of clinical studies is necessary. Because the results of the phase 4 studies are the proper criteria for how the vaccine works in the real world [5].

Vaccines have always been known to be the most effective and safest drugs; however, different side effects have been identified for them, for example, the link between influenza, hepatitis, and HPV vaccines with demyelinating syndromes has been discovered, and the injection of influenza vaccine is a reason for the incidence of narcolepsy in young people [6].

Because COVID-19 vaccines are urgently approved, meaning they do not complete the standard clinical trials, the adverse effects of each vaccine should be closely monitored. It is necessary to pay attention to the fact that in mass vaccination, due to different races, disease history, age, lifestyle, and other effective factors, the incidence of adverse effects of vaccination is higher. According to data from the CDC, VAERS, and EMA databases, the short-term outcome of COVID-19 vaccination is promising, but in the medium and long term, especially with some vaccines, side effects have been reported that are worrisome. VST is the most severe disorder that should be diagnosed and controlled immediately. Therefore, physicians and personnel of medical centers related to these patients should recognize these complications and intervene as soon as possible.

## Search method

**Fig. 1:**
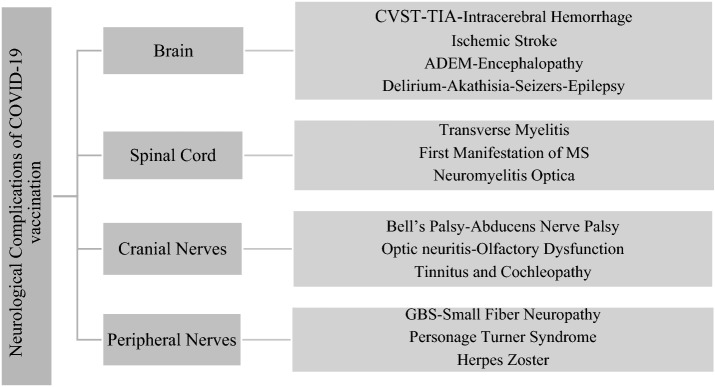
Classification of neurological complications observed after COVID-19 vaccination

Research, Review, and Case Report articles related to adverse effects of COVID-19 vaccination from 2020 to February 2022 were searched and reviewed in Google Scholar, PubMed, and NCBI databases. Many Case Report articles were not considered due to the lack of a convincing link between the complication and vaccination. Keywords used for this search included COVID-19, SARS-CoV-2, vaccination, side effects, complications, vascular thrombosis, thrombocytopenia, myelitis, demyelination, and all kind of mRNA vaccines, Adenovirus vaccine, Pfizer, AstraZeneca, Johnson & Johnson, Moderna, Sinovac, Sinopharm, Sputnik, and Covaxin. For ease of understanding the various side effects of COVID-19 vaccination, the main categories are shown in Fig. 1.

## Neurological complications following COVID-19 vaccination

According to reports published in the VAERS database, COVID-19 vaccines have several local and systemic neurological complications that occur in different people, from mild to severe, depending on age, sex, history of the disease, and pre-existing immunity [7]. Complications usually appear within one day to 1 month after injection and are usually acute, transient, and self-limiting, but in severe cases lead to hospitalization and intensive care [8]. On the other hand, women have the highest incidence of neurological complications because they induce a stronger immune response against foreign antigens, which can lead to the targeting of self-antigens and lead to autoimmune disorders [9]. Adverse reactions after the second dose of the vaccine are reported more than in the first dose [5].

Mild neurological effects of the COVID-19 vaccine include weakness, numbness, headache, dizziness, imbalance, fatigue, muscle spasms, joint pain, and restless leg syndrome are more common, while tremors, tinnitus, and herpes zoster are less common. On the other hand, severe neurological complications included Bell's palsy, Guillain–Barre syndrome (GBS), stroke, seizures, anaphylaxis, and demyelinating syndromes such as transverse myelitis and acute encephalomyelitis [10]. Among these, the most dangerous neurological complication caused by COVID-19 vaccines, especially adenovirus-based, is cerebral venous sinus thrombosis in women of childbearing age [8].

According to the WHO, in the case of side effects of inactivated virus-based vaccines, especially Sinopharm, the most common local and systemic adverse reactions are injection site reactions, fatigue, fever, headache, and allergic dermatitis, which are self-limiting, and the person does not need to be hospitalized [11, 12]. It is noteworthy that rare and scattered reports have been published on the side effects of Sinopharm and other inactivated virus-based vaccines (Table 1). Vaccine reactivity has been linked to a temporary increase in inflammatory cytokines that act on blood vessels, muscles, and other tissues. In other words, we will observe the flu-like syndrome for several consecutive days after vaccination [13]. According to a recent report on the Sputnik vaccine, side effects are included headache, joint pain, fever, and flu-like symptoms [14]. According to published information on the side effects of other adenovirus vaccines, it is essential to properly evaluate the efficacy of the Sputnik vaccine and publish relevant data to decide on its side effects. COVID-19 vaccination can sometimes have severe side effects on nervous system, including the brain, spinal cord, cranial nerves, and peripheral nerves, and has been shown to have adverse vascular, metabolic, inflammatory, and functional effects on the brain [1].

**Table 1:** Reported neurological complications for inactivated virus-based vaccines

Vaccine name	Complications	References
Inactivated virus		
Sinovac	Headache	[11, 12]
	Transverse myelitis	[15–18]
	Bell’s palsy	[19]
	Acute disseminated encephalomyelitis	[15, 20]
	neuromyelitis optica	[21]
Sinopharm	Multiple sclerosis relapse	[9, 22]
	Neuromyelitis optica	[21]
Covaxin	Bell’s palsy	[19, 23–25]

The two main mechanisms, ectopic immune reactions, and molecular mimicry, have been proposed for the pathogenicity of vaccines and how these complications occur.

## Headache

The first and most common systemic side effect of COVID-19 vaccines is headache, which is mild to severe and is felt in the frontal area of the head. Post-vaccination headaches can be caused by stress, vascular spasm, and intracerebral or subarachnoid hemorrhage. Vaccines based on mRNA and adenovirus have been reported to be most likely to cause headaches [26].

## Vascular complications in the brain

Due to the activity of the immune system, after the injection of COVID-19 vaccines, especially adenovirus-based type, thrombocytopenia, cerebral venous sinus thrombosis, ischemic stroke and intracerebral hemorrhage, have also been reported [27]. The proposed mechanism for thrombocytopenia is the synthesis of IgG antibodies against platelet factor 4 (PF4), which activates platelets and blood clots in large venous arteries [28]. Adenovirus-based vaccines are at the forefront of causing this complication due to the transfer of the nucleic acids encoding the viral spike (S) protein. Due to the leakage of these genetic materials and their binding to factor 4 platelet, autoimmunity develops [29]. Venous sinus thrombosis is associated with excessive coagulation. Vaccine viral antigens activate platelets or indirectly cause blood to clot by activating complement pathways and increasing thrombin production. Venous sinus thrombosis and cerebral hemorrhage are more common in women between the ages of 30 and 50 than in men (Table 2) [8].

**Table 2:** Reported neurological complications for adenovirus-based vaccines

Vaccine name	Complications	References
Adenovirus-based vaccine		
AstraZeneca	Headache	[8]
	Cerebral venous sinus thrombosis	[30–38]
	Transverse myelitis	[16–18, 39–44]
	GBS	[45–48]
	Bell’s palsy	[19, 23–25]
	Acute disseminated encephalomyelitis	[49]
	Intracerebral hemorrhage	[10, 50]
	Ischemic stroke	[19]
	Encephalopathy	[51]
	Parsonage–Turner syndrome	[52]
	Herpes zoster	[53]
	Tinnitus and cochleopathy	[54]
	Seizures	[10]
	Unilateral and bilateral optic neuritis	[10]
Johnson & Johnson	Cerebral venous sinus thrombosis	[31, 34, 38]
	Transverse myelitis	[16–18, 39]
	GBS	[55–58]
	Transient ischemic attack	[59]
Sputnik	Headache	[14]
	Multiple sclerosis	[60]

## Acute neurological disorders

These disorders include, transverse myelitis, acute diffuse encephalomyelitis (ADEM), Bell’s palsy, GBS, encephalopathy and seizures. Each type of vaccine can play a different role in increasing the risk of manifestation of these disorders (Tables 2, 3). The COVID-19 vaccine-related convulsions can be attributed to the synthesis and release of spike proteins, which cause severe inflammation and hyperthermia. Hyperthermia, in turn, increases glial cell activity and increases blood–brain barrier permeability. Following these events, as expected, peripheral blood cells and albumin enter the brain and disrupt the osmotic balance [10]. In connection with brain disorders, the possible mechanism is the entry of inflammatory mediators secreted by peripheral blood cells into the brain and the destruction of myelin and axonal degeneration. The presence of SARS-CoV-2 spike domain S1 antibodies in CSF may explain neurological complications after vaccination, such as encephalopathy and seizures [61].

**Table 3:** Reported neurological complications for mRNA-based vaccines

Vaccine name	Complications	References
mRNA-based vaccine		
Pfizer	Headache	[73, 74]
	First manifestation of multiple sclerosis	[75–77]
	Transverse myelitis	[78, 79]
	GBS	[55, 56, 80–83]
	Bell’s palsy	[19, 23–25, 84–86]
	Acute disseminated encephalomyelitis	[87]
	Neuromyelitis optica	[21, 76]
	Small fiber neuropathy	[64, 77]
	Encephalopathy	[10]
	Olfactory dysfunction and phantosmia	[66, 88]
	Tinnitus and cochleopathy	[89]
	abducens nerve palsy	[90]
	Parsonage–Turner syndrome	[1]
	Seizers	[10]
	Herpes zoster	[91, 92]
	Akathisia	[93]
	Delirium	[1]
	Intracerebral hemorrhage	[94]
	Ischemic stroke	[10, 95]
	Transient ischemic attack	[10]
Moderna	Transverse myelitis	[15, 17, 96]
	First manifestation of multiple sclerosis	[76, 94]
	Bell’s palsy	[19, 97]
	Encephalopathy	[98]
	Small fiber neuropathy	[77]
	Herpes zoster	[91, 99]
	Epilepsy	[100, 101]
	Intracerebral hemorrhage	[102]

Transverse myelitis is an inflammation of a part of the spinal cord that usually occurs after infection and is associated with impaired sensory, motor, and autonomic function (bladder and intestines) in areas below the area of inflammation in the spinal cord. The mechanism of induction of this disorder is the development of autoimmunity by molecular mimicry. In fact, the viral antigens of the vaccine stimulate an immunological response in the spinal cord [62]. Transverse myelitis has been observed after injection of mRNA and adenovirus-based vaccines, and it is noteworthy that mRNA-based vaccines can cause exacerbation or early manifestation of MS and neuromyelitis optica. More generally, the majority of demyelinating syndromes are related to mRNA-based vaccines, followed by adenovirus-based vaccines. According to reports, these complications are more common in men and women between the ages of 20 and 60 [9].

COVID-19 vaccination also affects the cranial and peripheral nerves and causes side effects such as Bell's palsy (facial nerve palsy—7 cranial nerve), abducens nerve palsy (lateral rectus ocular muscle nerve palsy—6 cranial nerve), impaired vision, olfactory, hearing, Guillain–Barre syndrome (GBS), small fiber neuropathy, Parsonage–Turner syndrome, and also herpes zoster. In this case, too, the known mechanism is the induction of autoimmunity by molecular mimicry. Bell's palsy and small fiber neuropathy are more commonly observed in mRNA-based vaccines [63, 64]. GBS is also a peripheral nerves and nerve roots injury that presents with severe motor weakness and paralysis of the legs or four limbs and is more common in the elderly after vaccination with adenovirus-based vaccines [65]. There have been many reports of the Pfizer vaccine being associated with olfactory [66], visual [67], auditory [68, 69], and sometimes abducens nerve palsy. Olfactory dysfunction ranges from a lack of sense of smell to an olfactory hallucination (phantosmia) that results from a bilateral disturbance or enhancement of the olfactory pathway and the olfactory bulb. Hearing disorders can vary from hearing loss to tinnitus and dizziness. Also, there is ample evidence that the Pfizer and AstraZeneca vaccines are associated with optic nerve inflammation and vision disorders and are more common in middle-aged people [70].

Herpes zoster is a disease that occurs as a result of the reactivation of the varicella-zoster virus (VZV) after receiving the COVID-19 vaccine. The process that causes the disorder is probably explained by the fact that the varicella-zoster virus CD8+ killer cells, after vaccination, are temporarily unable to control VZV due to the extensive change of simple CD8+ cells to the COVID-19 virus CD8+ killer cells. Therefore, vaccination is like a shock to the recurrence of VZV and subsequent herpes zoster [71]. mRNA-based vaccines can increase the risk of herpes zoster [72]. There was a recent report of Ramsey Hunt Syndrome (RHS after the Pfizer vaccination. RHS leads to facial nerve palsy, vestibulocochlear neuropathy, and glossopharyngeal nerve neuropathy, so it causes numbness of the face, tongue, and hearing loss. In addition, skin blisters have been observed in the ear area, leading us to hypothesize that reactivation of VZV could be a cause for RHS as well as Bell's palsy [71].

## Conclusion

According to the vaccine study literature, adverse effects have always been part of the mass vaccination strategy, but ultimately the desired effects of the vaccination are more significant. Side effects of COVID-19 vaccination have been reported more frequently in people with a history of immune-related diseases or who are more sensitive to age and physiological conditions. The most important and most common complications are cerebral venous sinus thrombosis (more about AstraZeneca), transverse myelitis (more about Pfizer, Moderna, AstraZeneca, and Johnson & Johnson), Bell's palsy (more about Pfizer, Moderna, AstraZeneca), GBS (more about Pfizer, AstraZeneca, and Johnson & Johnson), and the first manifestation of MS (more about Pfizer). Finally, discovering whether these disorders are accidental or whether the vaccine is the main cause of them requires future studies, ongoing efforts to gather evidence, and long-term monitoring.

## Data Availability

Not applicable.
